# Permafrost condition determines plant community composition and community‐level foliar functional traits in a boreal peatland

**DOI:** 10.1002/ece3.7818

**Published:** 2021-07-03

**Authors:** Katherine M. Standen, Jennifer L. Baltzer

**Affiliations:** ^1^ Department of Biology Wilfrid Laurier University Waterloo ON Canada

**Keywords:** carbon cycling, climate change, discontinuous permafrost, ecosystem function, environmental gradients, leaf economic spectrum, northwest territories, plant functional traits

## Abstract

Boreal peatlands are critical ecosystems globally because they house 30%–40% of terrestrial carbon (C), much of which is stored in permafrost soil vulnerable to climate warming‐induced thaw. Permafrost thaw leads to thickening of the active (seasonally thawed) layer and alters nutrient and light availability. These physical changes may influence community‐level plant functional traits through intraspecific trait variation and/or species turnover. As permafrost thaw is expected to cause an efflux of carbon dioxide (CO_2_) and methane (CH_4_) from the soil to the atmosphere, it is important to understand thaw‐induced changes in plant community productivity to evaluate whether these changes may offset some of the anticipated increases in C emissions. To this end, we collected vascular plant community composition and foliar functional trait data along gradients in aboveground tree biomass and active layer thickness (ALT) in a rapidly thawing boreal peatland, with the expectation that changes in above‐ and belowground conditions are indicative of altered resource availability. We aimed to determine whether community‐level traits vary across these gradients, and whether these changes are dominated by intraspecific trait variation, species turnover, or both. Our results highlight that variability in community‐level traits was largely attributable to species turnover and that both community composition and traits were predominantly driven by ALT. Specifically, thicker active layers associated with permafrost‐free peatlands (i.e., bogs and fens) shifted community composition from slower‐growing evergreen shrubs to faster‐growing graminoids and forbs with a corresponding shift toward more productive trait values. The results from this rapidly thawing peatland suggest that continued warming‐induced permafrost thaw and thermokarst development alter plant community composition and community‐level traits and thus ecosystem productivity. Increased productivity may help to mitigate anticipated CO_2_ efflux from thawing permafrost, at least in the short term, though this response may be swamped by increase CH_4_ release.

## Graphical abstract

Climate warming‐induced permafrost thaw may lead to changes in boreal peatland function through changes in plant community composition and function. Our results highlight that increasing depth to permafrost led to increases in plant community photosynthesis, respiration, specific leaf area, and foliar nitrogen as a result of a switch in community composition to more productive species. Ultimately, increased plant productivity may help to mitigate anticipated carbon dioxide release from thawing permafrost, at least in the short term, though this response may be overwhelmed by increased methane release.

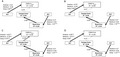

## 1 INTRODUCTION

Boreal peatlands store roughly 30%–40% of global terrestrial carbon (C) (Pan et al., 2011; Tarnocai et al., 2009), meaning that changes in boreal peatland C dynamics influence the global climate system (Lal et al., 2018; Roulet, 2000). C accumulation is high in these regions for many reasons. First, soils in boreal peatlands are cold and wet meaning that decomposition of plant litter is slow, which promotes the accumulation of deep organic soils (i.e., peat; Moore & Basiliko, 2006). Second, peat is often composed of *Sphagnum* mosses, which decompose slowly due to the chemical composition and structure of their tissues, further exacerbating the slow decomposition rates in peatlands (Moore & Basiliko, 2006; Rydin et al., 2006). Finally, many boreal peatlands are underlain by permafrost (perennially frozen ground; Jorgenson et al., 2006); because permafrost soils are at or below 0°C, decomposition is inhibited (Vardy et al., 2000). However, climate warming is leading to widespread increases in soil temperature and permafrost thaw in boreal peatlands (Biskaborn et al., 2019; Olefeldt et al., 2016), relaxing environmental constraints on decomposition. This has the potential to lead to a substantial release of carbon dioxide (CO_2_) and methane (CH_4_) to the atmosphere (Turetsky et al., 2019) resulting in a large, positive feedback to climate change.

In addition to altering boreal peatland C dynamics, permafrost thaw also leads to landscape‐scale changes, with both direct and indirect influences on plant community composition and plant functional traits (defined as characteristics of an individual plant related to its structure, physiology, or phenology; Violle et al., 2007). For example, permafrost thaw can result in ground subsidence and inundation, leading to wetland expansion at the expense of forest cover (i.e., lowland thermokarst; Baltzer et al., 2014) or changes to forest composition and structure (Dearborn et al., 2021). Such changes lead to a shift in understory plant community composition from slower‐growing evergreen shrubs to faster‐growing aquatic herbs (Camill, 1999; Camill et al., 2001). Large changes in plant community composition also influence functional traits relating to C uptake. Specifically, faster‐growing, more productive species like aquatic herbs tend to have greater foliar functional traits including greater gas exchange rates (photosynthesis and dark respiration), specific leaf area (SLA), and nitrogen (N) concentrations (e.g., Reich, 2014; Wright et al., 2004). As such, species turnover when coupled with changes in these foliar functional traits alter community‐level traits (sensu Roos et al., 2019) with potential implications for ecosystem C dynamics.

Warming‐induced permafrost thaw is also altering resource availability and, as such, may have both direct and indirect influences on plant community dynamics. Specifically, as permafrost thaws the active (seasonally thawed) layer thickens, locally increasing plant available N (via release of previously frozen N; Hewitt et al., 2019; Keuper et al., 2012; Salmon et al., 2016) and the volume of soil available for rooting. Given that the boreal biome is historically nutrient‐poor (Bonan & Shugart, 1989) and cold, shallow soils limit root function, these changes in the soil environment may directly increase plant productivity. For example, N tracer added at 40 cm soil depth has been detected in aboveground tissues of tundra species (Hewitt et al., 2019), with implications for plant functional traits. Increased N availability following permafrost thaw also drives changes in community composition: fast‐growing peatland species such as *Rubus chamaemorus* and the sedge tussock cottongrass (*Eriophorum vaginatum*) increased biomass with N amendment at depth (Keuper et al., 2017). Similarly, fertilization of subarctic communities tends to increase abundance of productive plant functional groups such as deciduous shrubs and graminoids at the expense of slower‐growing plant functional groups such as lichens (Haugwitz & Michelsen, 2011). On the other hand, nutrient increases may indirectly influence understory plant communities through changes in the overstory: N fertilization of black spruce (*Picea mariana*), a dominant boreal tree species, results in foliar trait changes including larger leaf area (Paquin et al., 1998), and greater foliar N (Johnsen, 1993; Paquin et al., 1998) and photosynthetic rate (Johnsen, 1993). Changes in the overstory may thus lead to cascading influences on the understory community through reduction of light on the forest floor (e.g., Marshall & Baltzer, 2015). As such, permafrost thaw, and associated increases in nutrient availability, may directly affect community‐level traits through intraspecific trait variation, species turnover, or both and indirectly by influencing the overstory thereby leading to increased competition for light between the under‐ and overstory.

Another important control on plant communities and traits in peatlands is organic layer thickness (OLT, or peat depth): thicker organic layers restrict access to more nutrient‐rich mineral soil and may have both direct and indirect influences on understory plant communities. Specifically, recruitment of trees in boreal peatlands tends to be higher (Préfontaine & Jutras, 2017) and basal area and species richness of trees and tall shrubs greater (Dearborn et al., 2021) when organic layer is thinner. Given these relationships and known links between nutrients and foliar traits, we expect that understory community‐level traits, through species turnover and/or intraspecific trait variation, will be directly influenced by access to more nutrient‐rich mineral soil. On the other hand, the greater basal area on thinner organic layers may correspond with greater canopy cover, thereby reducing light reaching the forest floor and leading to an indirect influence on understory community composition and traits. Indeed, greater overstory density decreases photosynthetic rates (Hébert et al., 2010) and leaf mass per area (Hébert et al., 2011) in the boreal evergreen shrub Labrador tea (*Rhododendron groenlandicum*). Thus, OLT, through access to more nutrient‐rich mineral soil, may directly influence traits and composition of understory plant communities or be indirectly influential via decreased light availability following changes in the overstory.

Importantly, rates of foliar gas exchange affect net primary productivity (NPP) of an ecosystem (e.g., Reich, 2012); as such, changes in community‐level foliar traits due to thaw‐induced environmental change may alter peatland C dynamics. Understanding the relative influences of understory community composition and intraspecific variation on community‐level traits in response to permafrost thaw is needed to better forecast future functioning of boreal peatland sites (e.g., Frolking et al., 2011). Although permafrost thaw is expected to cause an efflux of C to the atmosphere, increasing plant productivity following thaw may help mitigate this loss in the short term (as suggested by Helbig, Chasmer, Desai, et al., 2017; Keuper et al., 2017) through changes in plant community composition to more productive species with a faster suite of functional traits relating to C dynamics. However, changes in plant communities following thaw are unlikely to mitigate C loss in the long‐term (Abbott et al., 2016) especially given the large release of CH_4_ expected with increased wetland formation (Helbig, Chasmer, Kljun, et al., 2017).

**FIGURE 1:**
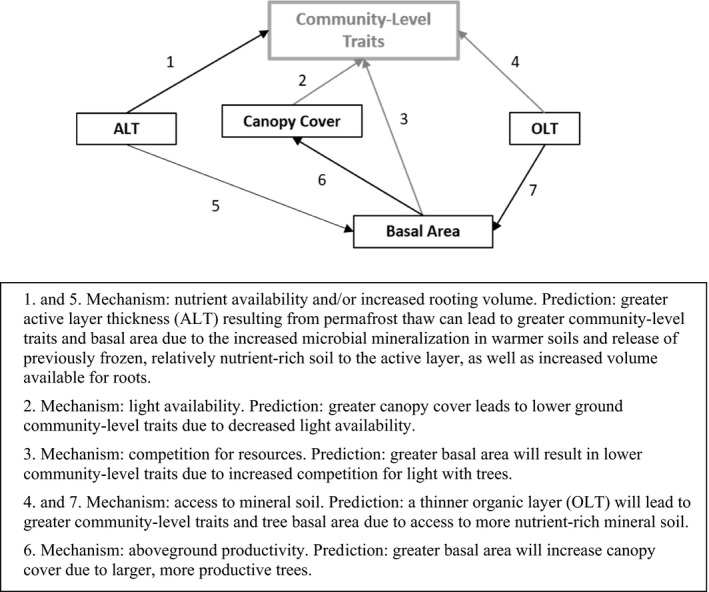
Conceptual model of hypothesized connections, and main mechanisms and predictions, between abiotic and biotic variables and their influence on community‐level plant functional traits of understory vegetation. Black and gray lines represent hypothesized positive and negative relationships, respectively. Predictions can occur because of intraspecific trait variability and/or species turnover

To better understand the implications of permafrost thaw‐induced environmental changes on ground vegetation communities, we collected data on understory community composition and foliar functional traits along a gradient in aboveground tree biomass and active layer thickness (ALT) at a boreal peatland site experiencing rapid and accelerating permafrost thaw (Baltzer et al., 2014). Our first objective was to determine the influence of key environmental variables, including ALT, canopy cover, tree basal area, and OLT, on plant community composition, as well as whole‐community foliar functional traits related to the C dynamics of understory vegetation. We tested the hypothesized connections between these abiotic and biotic factors and their direct and indirect influence on community‐level functional traits using Figure 1 as a framework. Secondly, we aim to determine whether changes in community‐level traits across these gradients in aboveground tree biomass and ALT are dominated by intraspecific trait variability, species turnover, or both. Through these objectives, we will be able to better infer the direct and indirect mechanisms by which ongoing permafrost thaw will impact plant community dynamics and ecosystem functioning (i.e., C cycling) of a high latitude boreal peatland. Since permafrost peatlands are a common feature in the boreal biome with fairly predictable plant community composition and response to thaw, understanding changes in plant productivity following thaw at this site could help inform peatland C dynamics in a changing climate with global implications.

## 2 METHODS

### 2.1 Site description

Our study was conducted within the Scotty Creek Forest Dynamics plot (61°18′ N, 121°18′ W), located in the headwater portion of the Scotty Creek basin approximately 50 km south of Fort Simpson, Northwest Territories, Canada (Appendix 1, Figure S1). The Scotty Creek basin is a peatland site in the sporadic discontinuous permafrost zone, and its headwater portion is characterized by raised forested peat plateaus underlain by permafrost (henceforth “forested plateaus”), where the water table closely follows the frost table (Quinton & Baltzer, 2013). Interspersed among forested plateaus are treeless or sparsely treed, permafrost‐free wetlands formed following thermokarst (i.e., ground surface subsidence) ranging from ombrotrophic bogs to nutrient‐rich fens with various levels of connectivity and surficial water table (henceforth “permafrost‐free peatlands”). Forested plateau vegetation is dominated by black spruce and Labrador tea, while species common to permafrost‐free peatlands where we sampled included *Menyanthes trifoliata*, *Maianthemum trifolium,* and graminoids such as *Eriophorum vaginatum* and many other sedges. Mosses include feathermosses (i.e., *Hylocomium splendens* and/or *Pleurozium schreberi*) and acrocarpous mosses (e.g., *Dicranum* spp.) on drier forested plateaus and *Sphagnum* spp. in permafrost‐free peatlands (Garon‐Labrecque et al., 2015). From the period of 1970–2010, ~11% of forest was lost at this site as a result of permafrost thaw and subsequent thermokarst (Carpino et al., 2018) with a mean annual rate of 0.26% between 1977 and 2010 (Baltzer et al., 2014). However, rates of forest loss are accelerating and between 2000 and 2010, occurred at about 0.45% per year (Baltzer et al., 2014). Mean summer (May–August) temperature, annual air temperature, and annual precipitation at Fort Simpson are 14.0°C, −2.8°C, and 387.6 mm, respectively (1981–2010; Environment and Climate Change Canada). Mean annual air temperature at Fort Simpson has increased by ~2.8°C since 1981 (Quinton et al., 2019).

The Scotty Creek Forest Dynamics plot is part of the Smithsonian Institute's Forest Global Earth Observatory (ForestGEO) program (Anderson‐Teixeira et al., 2015; Davies et al., 2021). The plot is 10 hectares laid out in 240, 20 m × 20 m grid cells, where all stems with a diameter at breast height (DBH; 1.3 m) greater than 1 cm have been identified to species, mapped, and measured for DBH. The plot captures a gradient of OLT (from about 40 cm to >2 m) and humification, which corresponds with aboveground tree biomass. ALT varies substantially throughout the plot, from about 40 cm to permafrost‐free. See Dearborn et al. (2021) for further details on the Forest Dynamics plot.

### 2.2 Community composition

To assess plant community compositional differences across the Scotty Creek Forest Dynamics plot, 40 grid cells were selected via stratified random sampling, where 10 grid cells were selected within each of four aboveground tree biomass categories (henceforth “tree biomass categories”): three categories on forested plateaus included high (>15 trees with a DBH >10 cm), medium (5–15 trees with a DBH >10 cm), and low (<5 trees with a DBH >10 cm) aboveground tree biomass, in addition to none (treeless permafrost‐free peatlands). These categories, based on the number of trees >10 cm DBH per grid cell, correspond to differences in ALT, OLT, and humification, and represent increasing basal area from permafrost‐free peatlands to the high tree biomass category. Thus, these tree biomass categories act as a proxy for differences in nutrient and light availability. In each selected grid cell, two, 1 m^2^ quadrats were randomly placed and all vascular plants were identified to species or genus in the case of some *Carex* spp., and stem counts were conducted to evaluate absolute abundance in late June of 2016 for the forested plateaus and June 2019 for the permafrost‐free peatlands. For consistency, quadrats in permafrost‐free peatland areas were placed exclusively in lawns, which are relatively flat expanses of moss (often *Sphagnum* spp.) of fens and bogs. Quadrat‐level estimates of community composition were averaged to represent the community of the entire grid cell.

### 2.3 Functional traits

We collected trait data for those vascular species that cumulatively comprise ≥75% of individuals across grid cells. These species belonged to several plant functional groups including deciduous and evergreen shrubs, forbs, graminoids, coniferous trees, and fern‐allies (i.e., pterophytes) (Appendix 1, Table S1) in July 2017 for the forested plateaus and July 2019 for the permafrost‐free peatlands. Though mosses, especially *Sphagnum* spp., are incredibly important to ecosystem functioning in boreal peatlands (Turetsky et al., 2010), we have excluded them from our study due to constraints related to measuring gas flux of moss from saturated systems (i.e., permafrost‐free peatlands), which represent 25% of our study areas, with the instrument available for use. Traits of interest are linked with plant productivity and include those relating to the leaf economics spectrum (Wright et al., 2004): mass‐corrected maximum photosynthetic rate (A_mass_), dark respiration rate (R_mass_), foliar nitrogen (N_mass_), and specific leaf area (SLA). We collected trait data for at least two grid cells in each of the four tree biomass categories to capture site‐wide variability. Within each of these grid cells, functional trait data were collected for three replicates of each species (Appendix 1, Table S1). Gas exchange was measured on leaves of each species using a LI6400XT open‐path portable gas exchange system (LI‐COR Biosciences Inc., Lincoln, Nebraska), equipped with a LED light source (LI6400‐02B). For all measurements, sample chamber CO_2_ concentrations were maintained at 400 µmol CO_2_ mol^‐1^ and chamber humidity ranged from 30% to 60%. Gas exchange was measured at two photosynthetic photon flux densities: 0 µmol/m^2^ s^−1^ (R_area_) and 1,500 µmol/m^2^ s^−1^ (A_area_). Sampled leaves were collected for determination of N_mass_ and SLA and placed in a sealed plastic bag for transport. Fresh leaves were scanned within 2–3 hr of collection and measured for area using ImageJ (Schneider et al., 2012). In the case of species where leaves did not fill the area of the chamber (e.g., *Vaccinium vitis‐idaea* and *V. oxycoccos*), this fresh leaf area value was used to correct the fluxes automatically produced by the LI6400XT. Leaves were then dried at 50°C for 5 days and weighed to an accuracy of 0.0001 g to calculate SLA as the ratio between fresh area and dry mass. The inverse of SLA (leaf mass area) was used to convert area‐based measurements to mass‐based measurements. Dried leaf samples were ground with a ball mill grinder and analyzed for N_mass_ using a 2,400 Series II CHNSO Elemental Analyzer (PerkinElmer) with an acetanilide standard and an accuracy of <0.3%.

### 2.4 Environmental variables

To evaluate drivers of plant community composition and functional trait data, we collected environmental data (e.g., ALT, OLT, and canopy cover) at each of the 80, 1 m^2^ quadrats in late‐August 2017 for the forest plateaus and mid‐July 2019 for the permafrost‐free peatlands. We made four replicate measures of late season (late‐August) ALT at each quadrat as depth to refusal of a 1.5 m metal rod. We measured OLT, which equates to peat depth across our study site, within 2 m of the southern edge of each quadrat by digging a small pit (not exceeding 1.5 m in depth for practical reasons) and measuring the depth to mineral soil or late season frost table. In some cases, ALT exceeded the 1.5 m limit of our probe, thus we categorized ALT as “shallow” <0.50 m, “medium” between 0.5 and 1.5 m and “deep” beyond the limits of our instrument (>1.5 m). Since OLT measurements also had a 1.5 m limit, this variable was categorized in the same way. To estimate cover of tall shrubs (i.e., >1 m tall) and trees, we used a densiometer at a height of 1 m to measure canopy openness at four locations (along the edge of each 1 m^2^ quadrat). These four values were averaged and converted to percent cover for each quadrat.

We determined stand structure (i.e., stem density and species composition) for all trees and large shrubs of DBH >1 cm across each grid cell from the existing forest dynamics plot data. Using these data, we calculated basal area as:

(1) $$\mathrm{BA}=\sum \left(\pi ({\frac{{\mathrm{DBH}}_{i}}{2})}^{2}\right)$$

of all tree stems, *i*, per grid cell. We could not calculate stem density and basal area for two grid cells because they were outside the mapped portion of the FDP; however, these grid cells were in permafrost‐free peatlands with negligible tree presence. Because basal area is calculated at the grid cell level, environmental variables measured at the quadrat scale (i.e., ALT, OLT, and canopy cover) were averaged to represent the environment of the entire grid cell.

### 2.5 Statistical analysis

We used *R* v. 3.6.1 (R Core Team, 2019) to conduct all statistical analyses, and *ggplot2* (Wickham, 2016) and *ggpubr* (Kassambara, 2018) to create figures. To determine the influence of environmental variables on plant community composition across the Scotty Creek Forest Dynamics plot, we ran ordinations using the *vegan* package (Oksanen et al., 2018). Specifically, we used a Bray–Curtis dissimilarity matrix on Hellinger‐transformed stem count data and conducted a principal coordinate analysis (PCoA). Next, we conducted a redundancy analysis (RDA) on Hellinger‐transformed stem count data and standardized (mean = 0, standard deviation = 1) environmental variables, and used variance inflation factor (VIF) to assess whether environmental variables were collinear. We considered variables collinear if the VIF score was greater than 5 (Hair et al., 2006). Although OLT and ALT were moderately correlated (*r* = .48), the VIF score of these variables was low (<3), and thus, both were retained in the analysis. Basal area and stem density, on the other hand, were strongly correlated (*r* = .79), and thus, we removed stem density from further analyses because we felt that basal area better represented the aboveground biomass of trees than stem density. We also tested significance and overall fit of the RDA model and determined the significance of each axis and each environmental variable in the RDA.

To assess the extent of intra‐ and interspecific functional trait variability, we standardized all traits (mean = 0, standard deviation = 1) and calculated the interspecific variation of each trait as the variance across the means of each species. We then calculated intraspecific variability of each trait as the variance across all individuals of each species and within‐plant functional group variability as between‐species variance in that plant functional group.

To determine community‐level traits for each grid cell, we computed community‐weighted means (CWMs) across our plant communities for four traits involved in carbon cycling (A_mass_, R_mass_, N_mass_, and SLA) as:

(2) $${\mathrm{CWM}}_{\mathrm{tp}}=\sum_{i=1}^{S}\;a_{\mathrm{ip}}\times t_{i}$$

where *a_ip_* is the abundance (stem counts) of species *i* in sites *p,* and *t_i_* is the mean trait value of each species (Garnier et al., 2004; Muscarella & Uriarte, 2016). We used specific‐trait means which average trait values of all individuals of a species within each of four tree biomass categories giving a maximum of four mean trait values per species Thus, more abundant species contribute more to community‐level traits than less abundant species. We used ANOVA to assess differences in CWMs of each trait among tree biomass categories, and in the case of significant predictors, we used Tukey's HSD test for post hoc comparisons.

Finally, we determined connections among environmental variables and their influence on community‐level traits by testing the hypotheses presented in Figure 1 using piecewise structural equation models (SEM) of standardized (mean = 0, *SD* = 1) CWM traits and environmental variables in the *piecewiseSEM* package (Lefcheck, 2016). We fit SEMs for each CWM trait (A_mass_, R_mass_, N_mass_, and SLA) using linear regressions. Goodness of fit was determined using Fisher's *C* statistic for the whole model as well as the R^2^ of each individual model within the SEM. In the case of a significant relationship between CWMs and either categorical variable (i.e., ALT or OLT), we used one‐way ANOVA and Tukey HSD to evaluate differences in community‐level traits among these categories. Assumptions of ANOVA and linear regression were evaluated visually and met.

## 3 RESULTS

### 3.1 Community composition

**FIGURE 2:**
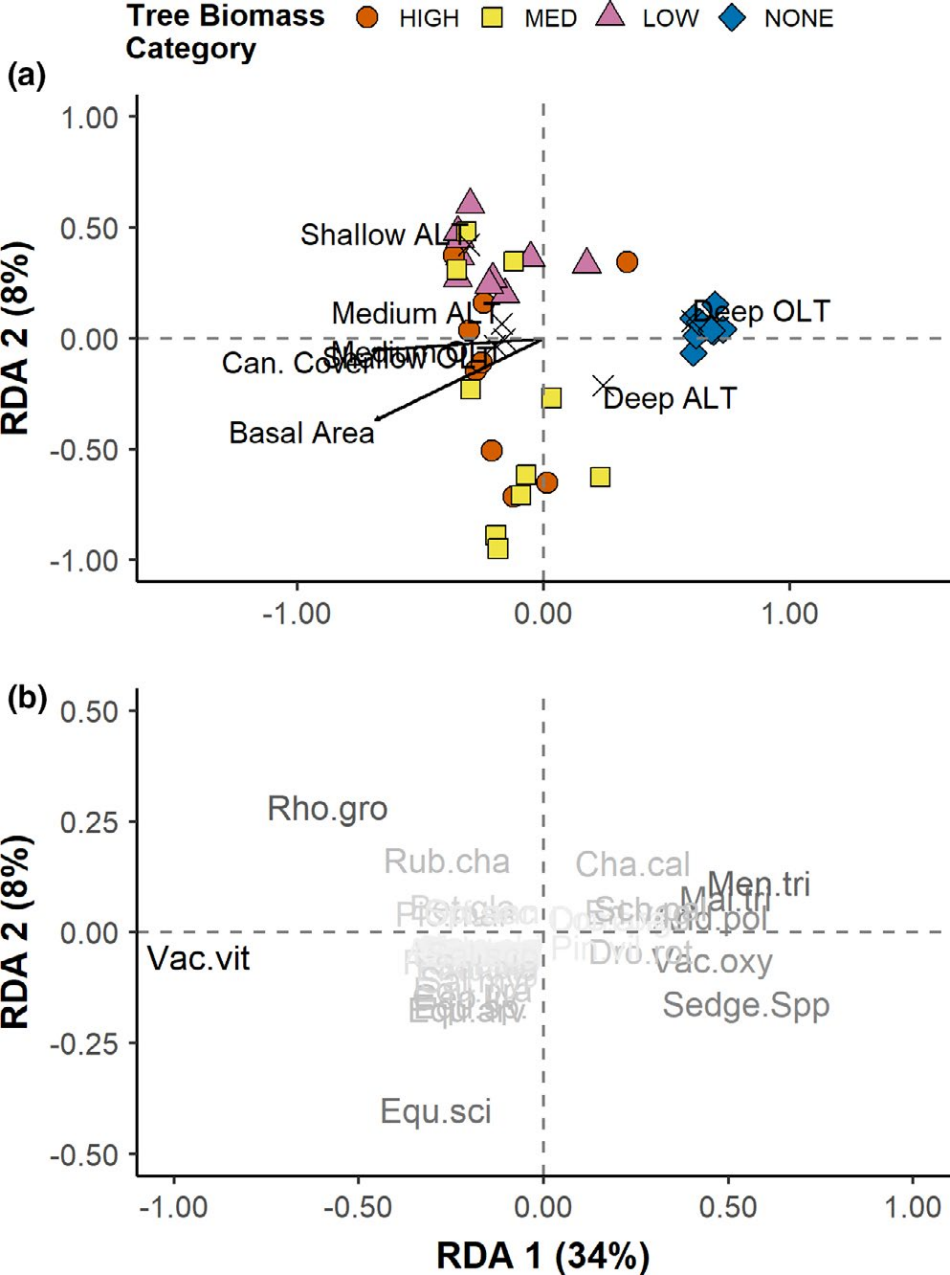
Redundancy analysis of the effect of four environmental variables on understory plant community composition across the Scotty Creek Forest Dynamics plot showing site scores and environmental variables (a) and species scores (b). Black arrows and X's represent scores of continuous and categorical variables, respectively. OLT = organic layer thickness, ALT = active layer thickness, Can. Cover = % canopy cover. Refer to Appendix 1, Table S1 for species codes

Plant community composition varied among the four tree biomass categories (Figure 2; Appendix 1, Figure S2), and the PCoA (Appendix 1, Figure S2) explained about 37% of variation in community composition. The first PCoA axis explained the majority of community compositional variation (29%) and represented a gradient from permafrost‐free peatlands to forested plateaus. As such, the differences in community composition are likely driven by the contrast between surficial water table in the permafrost‐free peatlands to water table that closely tracks ALT on the forested plateaus (Quinton & Baltzer, 2013). Specifically, permafrost‐free peatlands separated completely from all forested plateau areas and were associated with greater abundance of sedges and forbs such as *Menyanthes trifoliata* and *Maianthemum trifolium*. Tree biomass categories on forested plateaus did not differentiate across the first PCoA axis. However, along the second PCoA axis, which explained only 8% of variation, medium tree biomass separated from low tree biomass areas and tended to be associated with pterophytes such as *Equisetum scirpoides* and *E. arvense*, whereas low tree biomass areas were more associated with a greater abundance of evergreens such as *Vaccinium vitis‐idaea* (an ericoid shrub) and black spruce (*Picea mariana*), the forb *Rubus chamaemorus,* and the deciduous shrub *Betula glandulosa*. Plant community composition in high tree biomass areas overlapped with both low and medium tree biomass regions.

Active layer thickness and OLT were significant predictors of community composition across our study site (Figure 2). General patterns of distribution of each sampled community (i.e., “site scores”) and associations of these communities with plant species in the RDA were similar to that in Figure S2 (Appendix 1), suggesting that the predictors were a good fit for the gradient in community composition across the site. The overall analysis was significant (Appendix 1, Table S2), and the fit of the model was moderate (*R* ^2^_adj_ = 0.38), suggesting that the measured environmental variables were important in determining community composition. An ANOVA (Appendix 1, Table S3) showed that the first two axes were significant, and the RDA showed that these axes explained 34 and 8%, respectively. In addition, applying ANOVA to each predictor in the RDA model showed that OLT and ALT were significantly influencing community composition (Appendix 1, Table S4). OLT was closely associated with axis 1 and was greatest in permafrost‐free peatlands, suggesting that greater OLT was associated with greater abundance of forbs and sedges. Shallow and medium OLT were associated with greater abundance of evergreen shrubs and the forb *R. chaemamorus*. Greater ALT was equally associated with axis 1 and 2 and greater abundance of aquatic forbs, sedges, and horsetail species, whereas shallow and medium ALT tended to be more associated with greater abundance of evergreen shrubs and *R. chaemamorus*. Finally, basal area and canopy density, though not significant, primarily influenced RDA axis 1 and were positively associated with abundance of evergreen shrubs.

### 3.2 Community‐level plant functional traits

**FIGURE 3:**
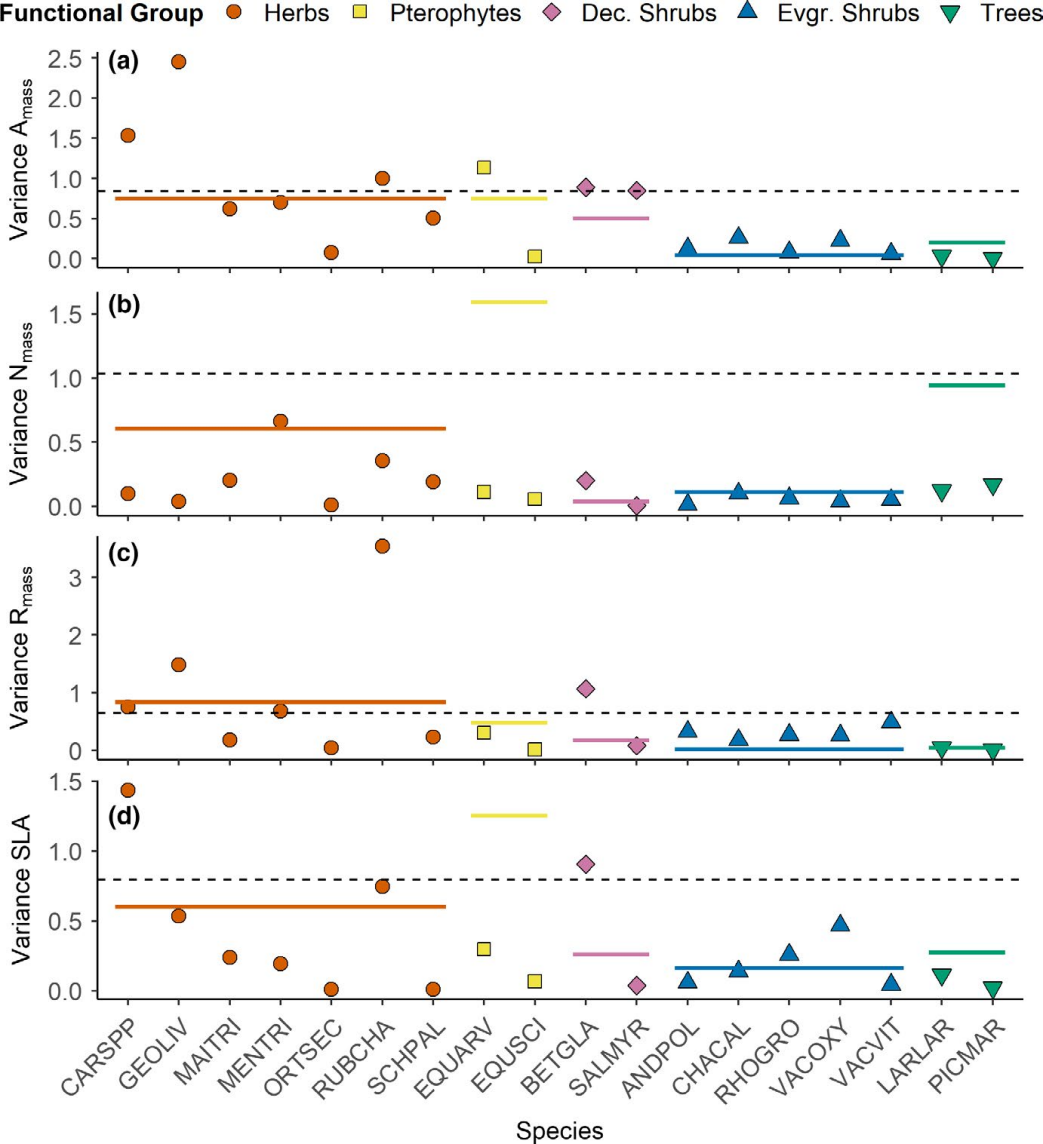
Interspecific (dashed gray line), intraspecific (colored points), and within‐plant functional group (colored bars) variability (as variance) of four leaf economic traits: mass‐corrected maximal photosynthetic rate (a), foliar nitrogen (b), dark respiration rate (c), and specific leaf area (d) across the Scotty Creek Forest Dynamics plot. Species codes listed in Appendix 1, Table S1

Interspecific variation exceeded intraspecific variation in most species‐trait combinations (Figure 3). In general, herbaceous species tended to have greater intra‐ than interspecific variability compared to other functional groups; however, this is only true for A_mass_ (Figure 3a) and R_mass_ (Figure 3c) and for less than half of herbaceous (both forbs and graminoids) species. Intraspecific variability of the pterophytes generally was less than interspecific variability for all traits. For deciduous shrubs, intraspecific variability was greater than interspecific variability for R_mass_ (Figure 3c) and SLA (Figure 3d) in *B. glandulosa* whereas *Salix myrtillifolia* did not exhibit greater intraspecific variation for any trait. In contrast, intraspecific variability of evergreen shrubs and trees never exceeded interspecific variability for any measured trait.

**FIGURE 4:**
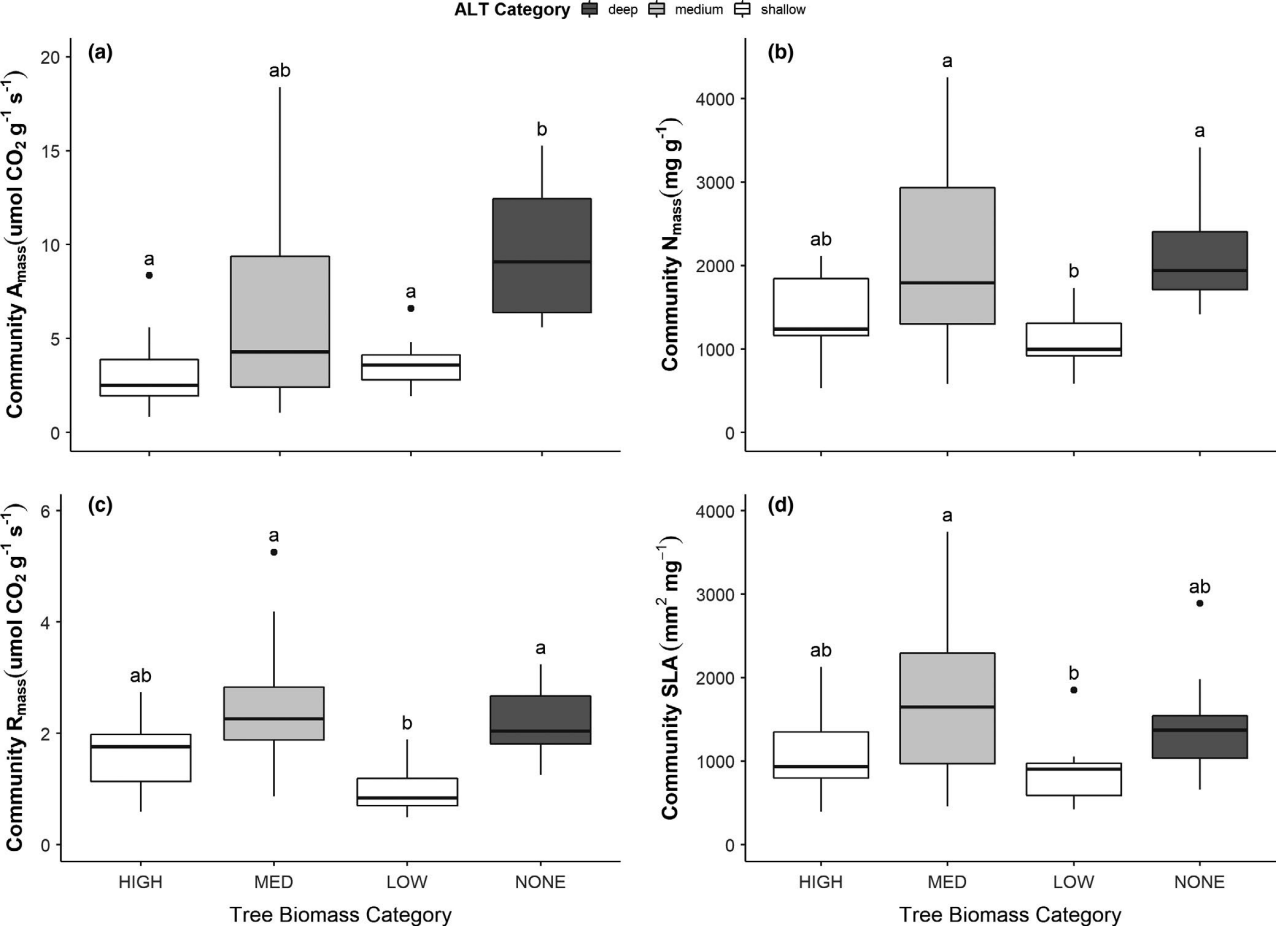
Boxplots showing variability within and differences among tree biomass categories for community‐weighted mean photosynthetic rate (a), foliar nitrogen (b), dark respiration rate (c), and specific leaf area (d). Different letters denote significant (*p* < .05) differences between categories. Bars are filled according to average active layer thickness (ALT) of that tree biomass category

Community‐level traits varied significantly across the tree biomass categories (Figure 4; Appendix 1, Table S5). In general, CWM traits were significantly greater in treeless permafrost‐free peatlands than in low tree biomass areas and in the high tree biomass areas in the case of community‐weighted A_mass_ (Figure 4a). However, in general permafrost‐free peatlands (i.e., no tree biomass) and medium tree biomass areas tended to have greater community‐level traits than either low or high tree biomass areas.

**FIGURE 5:**
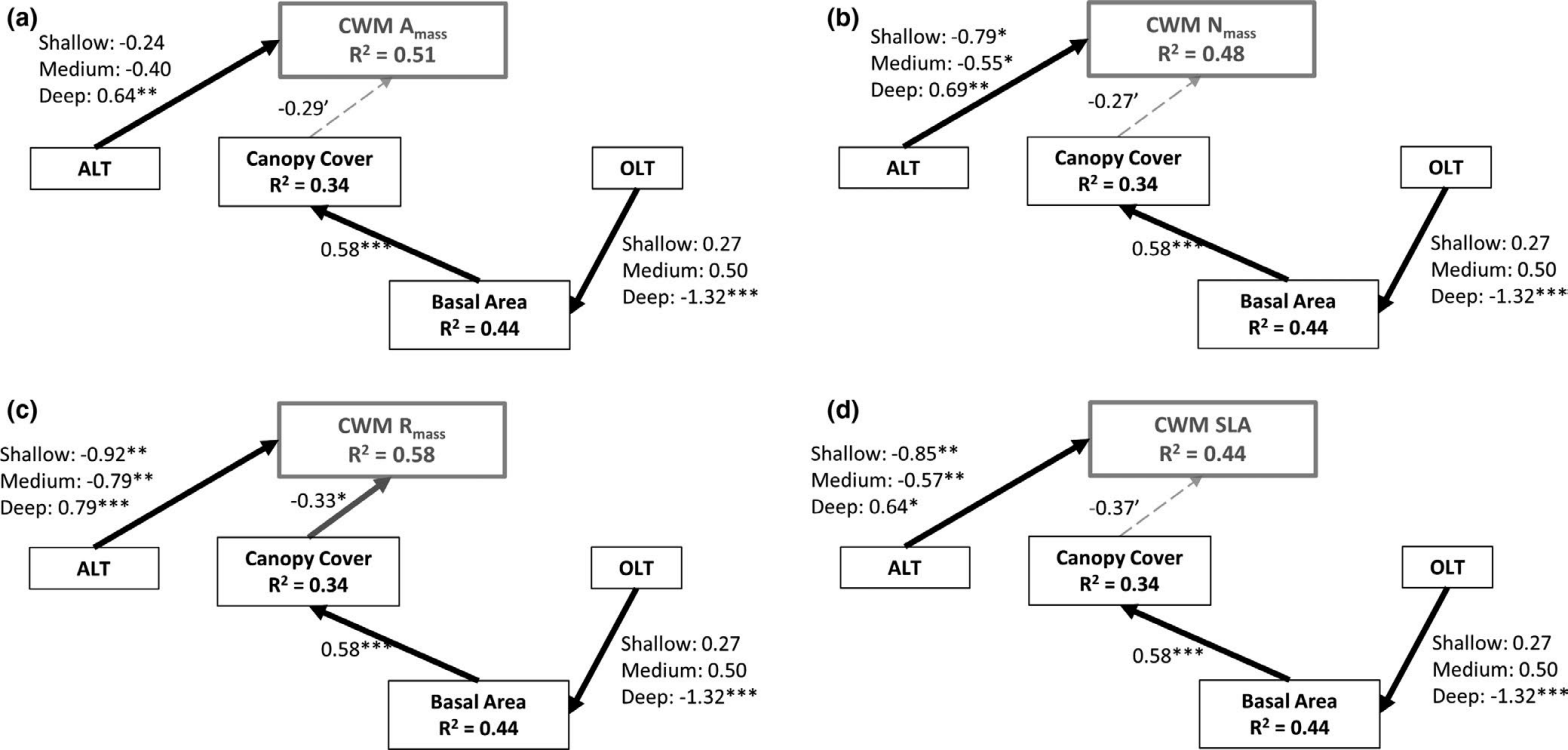
Structural equation models demonstrating the influence of environmental variables (black boxes) on community‐level plant functional traits (gray boxes) as community‐weighted mean mass‐corrected photosynthetic rate (a), foliar nitrogen (b), dark respiration rate (c), and specific leaf area (d). Black and gray solid lines represent significant positive and negative relationships, respectively, dashed lines represent marginally (*p* < .10) significant pathways, and nonsignificant pathways were removed (see Figure 1 for hypothesized relationships). Numbers associated with lines represent estimates for continuous and categorical variables. Response and predictor variables were standardized to be on the same scale for ease of interpretation. Fisher's C statistic for each model was *p* = .264, meaning our model is a good representation of the data and that no additional pathways would improve model results. ALT = active layer thickness and OLT = organic layer thickness. '*p* < .10, *p < .05, **p < .01, ***p < .001

The observed differences in community‐level traits were primarily driven by ALT, with greater ALT leading to greater community‐level traits (Figure 5). Specifically, SEM results of CWM A_mass_ (Figure 5a), N_mass_ (Figure 5b), R_mass_ (Figure 5c), and SLA (Figure 5d) showed that ALT was a direct significant, positive predictor of community‐level traits as hypothesized in Figure 1. In contrast to our predictions, basal area was not a significant predictor of community‐level traits; however, canopy cover showed the expected negative relationship with CWM traits but significantly so only for R_mass_ where increasing canopy cover reduced community respiration. Though only marginally significant (*p* < .1), CWM SLA, A_mass,_ and N_mass,_ also tended to decrease with canopy cover (Figure 5). Counter to our expectations, OLT did not directly influence CWM traits; thus, access to more nutrient‐rich mineral soil was not a determinant of understory community‐level traits. However, OLT did have a significant, negative relationship with basal area, suggesting that tree biomass was impacted by access to mineral soil. At the same time, basal area had a significant, positive relationship with canopy cover, suggesting that OLT indirectly influenced canopy cover via its role in promoting tree basal area production. Taken together, these results suggest that OLT is indirectly influencing CWM traits via influences on overstory structure. All four models of CWM traits had moderate fits and explained between 44% and 58% of total CWM trait variation. Basal area explained about 34% of variation in canopy cover and combined, ALT and OLT explained about 44% of variation in basal area.

**FIGURE 6:**
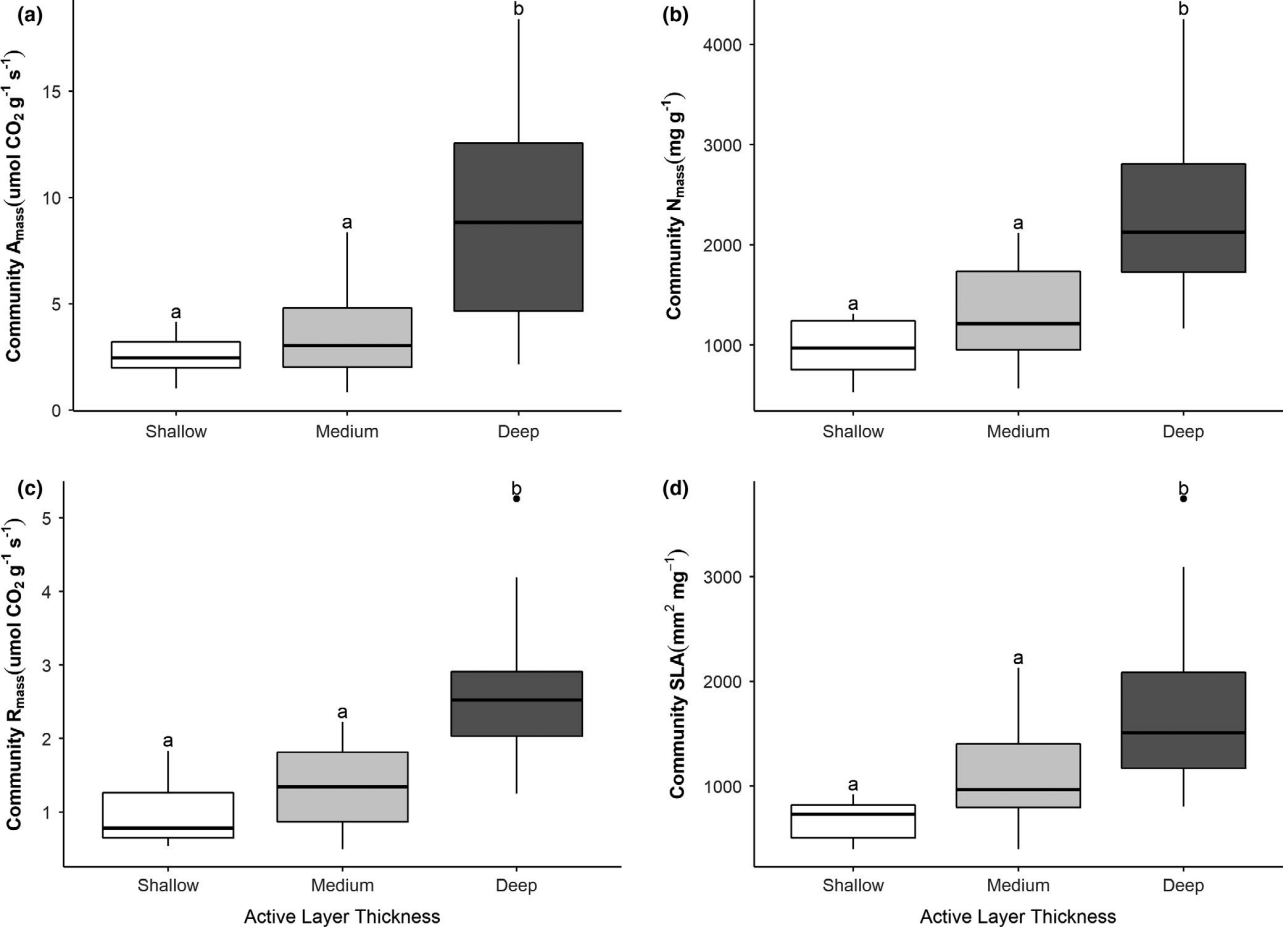
Boxplots showing variability within and differences among active layer thickness categories for community‐weighted photosynthetic rate (a), foliar nitrogen (b), dark respiration rate (c), and specific leaf area (d). Different letters denote significant (*p* < .05) differences between categories

To explore differences in community‐level traits with ALT, we ran one‐way ANOVAs and Tukey's HSD test on each CWM traits based on the ALT categories (Figure 6; Appendix 1, Table S6). All models were significant (*p* < .05; Appendix 1, Table S6) and showed that sites with deep active layer (>1.5 m) had significantly higher CWM traits than either the shallow (<0.5 m) or medium active layer (0.5–1.5 m) categories (Figure 6), suggesting greater community‐level traits (i.e., greater productivity) with thicker active layer.

## 4 DISCUSSION

Our results demonstrate that in a rapidly thawing boreal peatland, direct impacts of ALT were strongly influencing both plant community composition and community‐level traits. As such, ongoing permafrost thaw in this boreal peatland can be expected to have direct impacts on the functioning of the understory vegetation community. Specifically, greater ALT increased CWM A_mass_, R_mass_, N_mass_, and SLA, and also led to increased abundance of fast‐growing aquatic graminoid and forb species and horsetails and decreased abundance of slow‐growing evergreen shrubs as well as the forb *Rubus chamaemorus*. Moreover, variation in community‐level traits was likely driven by these large shifts in community composition and not intraspecific variation, which tended to be substantially less than interspecific variation. This means that species turnover was influencing CWM traits more than plastic trait responses to changing conditions. Though not as strong as ALT, OLT had an important but indirect role in influencing CWM traits through impacts on forest structure, highlighting the importance of OLT for forest structure and the cascading influence of light on understory community‐level traits. Ultimately, the changes in CWM traits with permafrost thaw and subsequent shifts in plant community composition documented at our rapidly thawing peatland site will play a key role in building understanding of the C cycle of boreal peatlands and informing accurate predictive models with ongoing climate change in Canada's north (e.g., Yu et al., 2011).

### 4.1 Influence of active layer thickness on community composition and traits

Plant community composition was strongly influenced by ALT: greater ALT was associated with more abundant faster‐growing graminoid and forb species at the expense of slower‐growing evergreen shrubs as well as the forb *Rubus chamaemorus*. Our results are comparable to other studies on the effects of permafrost thaw on community composition in boreal peatlands (e.g., Camill, 1999; Camill et al., 2001). Specifically, thaw can result in thermokarst and resulting collapse scars with simultaneous loss of or decrease in aboveground biomass, shifts away from peat plateau taxa such as black spruce and ericaceous shrubs, and increased importance of wetland‐adapted plants such as *Carex* spp. (Camill et al., 2001). Notably, Camill et al. (2001) found that forbs were almost entirely absent from these collapse scars in stark contrast to our results. However, Camill (1999) demonstrated variation in plant communities among different thaw features in boreal peatlands of Manitoba, Canada. For example, aquatic forbs such as *Menyanthes trifoliata*, which were common in permafrost‐free peatlands across our site, were abundant at the edges of collapse scars with more fen‐like characteristics (i.e., higher pH) and were absent from lower pH bog‐like systems (Camill, 1999). Thus, while the continued transition of forested plateaus underlain by permafrost to permafrost‐free peatlands at our site will lead to a community change from slower‐growing shrubs common and abundant on plateaus to faster‐growing wetland species, this will be mediated by groundwater inputs that shape the resulting vegetation communities (e.g., Bubier, 1995). Importantly, we have demonstrated that this shift in species composition will lead to marked transitions in community‐level traits.

Community‐level traits, as characterized by CWM A_mass_, R_mass_, N_mass_, and SLA, increased with ALT, in support of our hypothesis (Figure 1). This relationship was likely a result of shifts in community composition, and not intraspecific trait variability, which corresponded with the finding that interspecific variation exceeded intraspecific variation for most trait‐species combinations (Figure 3). Other studies have demonstrated similar patterns: Dwyer et al. (2014) found that, in an annual plant community, increased CWM SLA along a gradient of soil N was driven by community compositional shifts. Similarly, Roos et al. (2019) showed that species turnover was the main component of variation in community SLA, leaf dry matter content, foliar N, and pH of vascular plants across an altitudinal gradient. Though intraspecific variation has been demonstrated as important in some studies (e.g., Lepš et al., 2011; Roos et al., 2019) and is often thought to be more important at smaller spatial scales (Albert et al., 2011), the relatively low intra‐ compared to interspecific variation demonstrated herein may be attributable to strong environmental gradients present at our site. Along a relatively short distance (800 m), ALT ranged from only 0.4 m to greater than 1.5 m, basal area ranged from 0 to 25 m^2^ ha^−1^, and soil conditions varied from thick organic soils with surficial water table where permafrost was absent to relatively dry soils with variable ALT and OLT on forested plateaus. These gradients create diverse microhabitats that support distinct species assemblages with unique suites of traits.

### 4.2 Influence of other environmental variables on community composition and traits

In addition to ALT, OLT significantly affected plant community composition and had an indirect influence on CWM traits via changes in forest structure. Specifically, we found that a thicker soil organic layer promoted greater abundance of sedges and forbs. This relationship is likely because greatest OLT was observed in permafrost‐free peatlands where specialist aquatic species were more abundant. Thus, tolerance of saturated conditions in wetlands with deep organic soils is likely having the greatest influence on plant community composition (Camill, 1999). On the other hand, where the soil organic layer was thinner, slower‐growing evergreen shrubs were more abundant. This relationship was surprising, as we expected that more resource acquisitive species would be present where organic layer was thinner because of increased access to relatively nutrient‐rich mineral soils (e.g., Reich, 2014). Rather, increased abundance of evergreen shrubs with thinner organic layer could be in part a result of increasing basal area and canopy cover of trees: greater canopy closure associated with higher basal area requires greater tolerance of light limitation (Marshall & Baltzer, 2015). Because of lower light availability, plant species with more resource conservative strategies may be more abundant where OLT is thinner (Reich, 2014), a hypothesis partially supported by our SEM results. Indeed, OLT indirectly affected CWM traits via a direct, positive influence on basal area and an indirect effect on canopy cover. Canopy cover had a significant negative relationship with CWM R_mass_ and, though only marginally significant, also tended to have a negative influence on community A_mass_, N_mass_, and SLA.

### 4.3 Inter‐ versus intraspecific variability

Interspecific variability exceeded intraspecific variability in most species‐trait combinations. In general, boreal sites tend to have low nutrient availability (e.g., Hobbie et al., 2002) and short growing seasons (reviewed in Bonan & Shugart, 1989). Based on the fast‐slow continuum of plant strategies (Reich, 2014), slow‐growing species such as evergreen shrubs are more common and abundant, as these species are better equipped to persist under harsh conditions. However, evergreen shrubs also tend to be less plastic and thus less responsive to changing environmental conditions. On the other hand, with climate change and permafrost thaw, warming soils (e.g., DeMarco et al., 2014), increasing soil resource availability (e.g., Keuper et al., 2012, 2017), and lengthening growing seasons (e.g., Price et al., 2013), the more resource acquisitive strategy employed by forbs and graminoids may be favored, allowing these taxa to become more common and abundant. These fast‐growing plant functional groups also tend to have greater intraspecific variation than evergreen shrubs, as demonstrated herein (Figure 3) and by Wang and Moore (2014) and may thus be better equipped to respond to the rapidly changing environment. Consequently, as species composition continues to shift to faster‐growing species with warming and permafrost thaw, we may expect intraspecific variation to become increasingly important in peatland sites.

### 4.4 Implications of changing community traits for carbon cycling

The functional traits we considered are integral to understanding potential changes in C balance of boreal peatlands in several ways. First, an increase in CWM SLA with thickening active layers—due to the corresponding increase in dominance of graminoids and forbs—will lead to greater inputs of labile leaf litter (Santiago, 2007), resulting in faster decomposition of litter and greater CO_2_ efflux. Second, dominance of more productive taxa may increase root exudate inputs which prime soil microbes for decomposition (Wild et al., 2016). Lastly, these herbaceous species tend to have higher root turnover (Blume‐Werry et al., 2019), thus adding more root litter to the soil for decomposers. Combined with the release of temperature constraints on microbial activity as soils warm, we may expect decomposition rates of all plant litter to increase (Keuper et al., 2012; Salmon et al., 2016) resulting in a large CO_2_ efflux from the soil. Simultaneously, shorter‐lived roots tend to fall on the acquisitive side of the economic spectrum (Roumet et al., 2006) and thus may be better able to quickly access newly available nutrient pools. In addition, greater SLA facilitates greater CO_2_ uptake (e.g., Wright et al., 2004) and may also lead to faster nutrient cycling because of the accelerated decomposition associated with thinner leaves. Indeed, SLA is positively associated with A_mass_ and N_mass_ in both our study (*r* = .65 and *r* = .69, respectively, data not shown) and globally (Santiago, 2007; Wright et al., 2004). In this historically nutrient‐poor system (Bonan & Shugart, 1989), increasing N availability (Keuper et al., 2012; Salmon et al., 2016) and changing hydrological regime (O'Donnell et al., 2012) with permafrost thaw favors faster‐growing, more productive species as we and others (e.g., Camill, 1999) have demonstrated. However, we are likely underestimating community‐level functional traits at this site because we exclusively measured vascular species; mosses, especially *Sphagnum* spp. common in permafrost‐free peatland features at this site, are known to account for up to 50% of peatland NPP (Turetsky et al., 2010). Our findings suggest that increased plant productivity with thaw may mitigate these new sources of CO_2_ (as suggested by Keuper et al., 2017), at least in the short term. That being said, increased methane fluxes with warming may overwhelm this signal (Hanson et al., 2020) and plant biomass is unlikely to account for C loss in warming permafrost systems in the long term (Abbott et al., 2016).

## 5 CONCLUSIONS

In the boreal peatland studied herein, we demonstrated that community‐level plant functional traits (as CWM A_mass_, R_mass_, N_mass_, and SLA) of the vascular understory increase substantially in response to active layer thickening. Furthermore, we determined that these changes in CWM traits were most likely related to a switch in community composition from evergreen shrubs to aquatic herbaceous species in response to increasing ALT and the associated soil conditions of permafrost‐free parts of this landscape. We also found an indirect influence of OLT on CWM traits via modifications of forest structure and thus light availability. Combined, these findings help untangle the mechanisms driving ecosystem function changes in the face of rapid permafrost thaw and provide valuable process understanding to support modeling of boreal peatland functioning. Importantly, our findings that plant community productivity increase with permafrost thaw in a rapidly thawing boreal peatland as result of changing community composition suggests a possible mechanism for mediating some of the anticipated increase in CO_2_ efflux, at least in the short term. However, such changes could very well be overwhelmed by CH_4_ release, which is known to increase with warming (Hanson et al., 2020) and thermokarst development (Helbig, Chasmer, Kljun, et al., 2017).

## CONFLICT OF INTEREST

The authors declare no conflict of interest in this manuscript.

## AUTHOR CONTRIBUTIONS

**Katherine Marie Standen:** Conceptualization (equal); Data curation (lead); Formal analysis (lead); Investigation (lead); Methodology (lead); Visualization (lead); Writing‐original draft (lead); Writing‐review & editing (equal). **Jennifer Baltzer:** Conceptualization (equal); Formal analysis (supporting); Funding acquisition (lead); Methodology (supporting); Resources (lead); Supervision (lead); Visualization (supporting); Writing‐original draft (supporting); Writing‐review & editing (equal).

## DATA AVAILABILITY STATEMENT

The data included in this manuscript are accessible through the Wilfrid Laurier University Library Research Data Repository Dataverse (https://doi.org/10.5683/SP2/R4FTPW).

## Supporting information

Fig S1

Fig S2

Tab S1‐S6
