# Supplementary figures and images for: Permafrost condition determines plant community composition and community‐level foliar functional traits in a boreal peatland
Source: Ecol Evol. 2021 Jul 3;11(15):10133–46. doi: 10.1002/ece3.7818 (PMC8328418; doi:10.1002/ece3.7818)

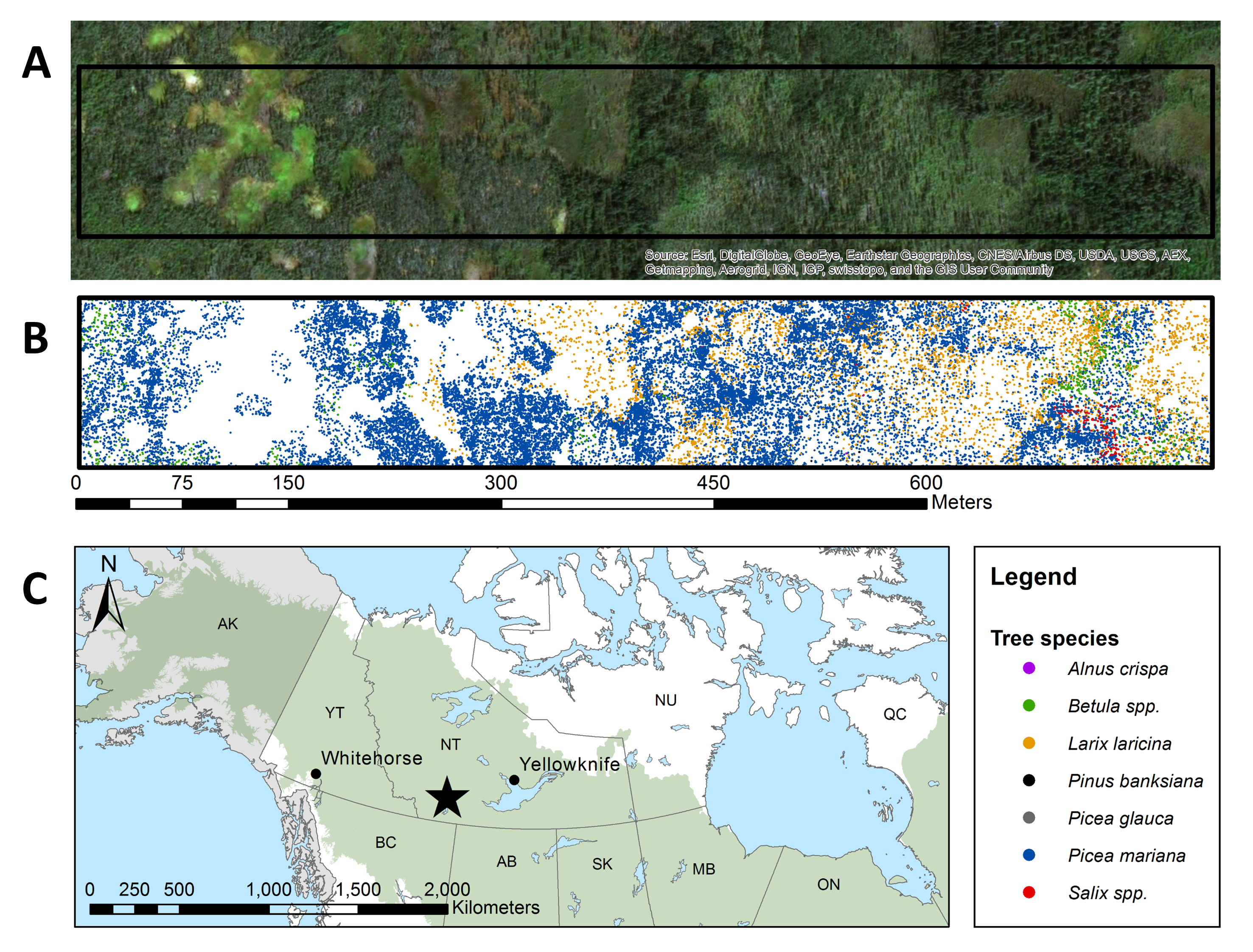

Supplement: Supplementary file 1 — Fig S1 [file ECE3-11-10133-s002.tif]

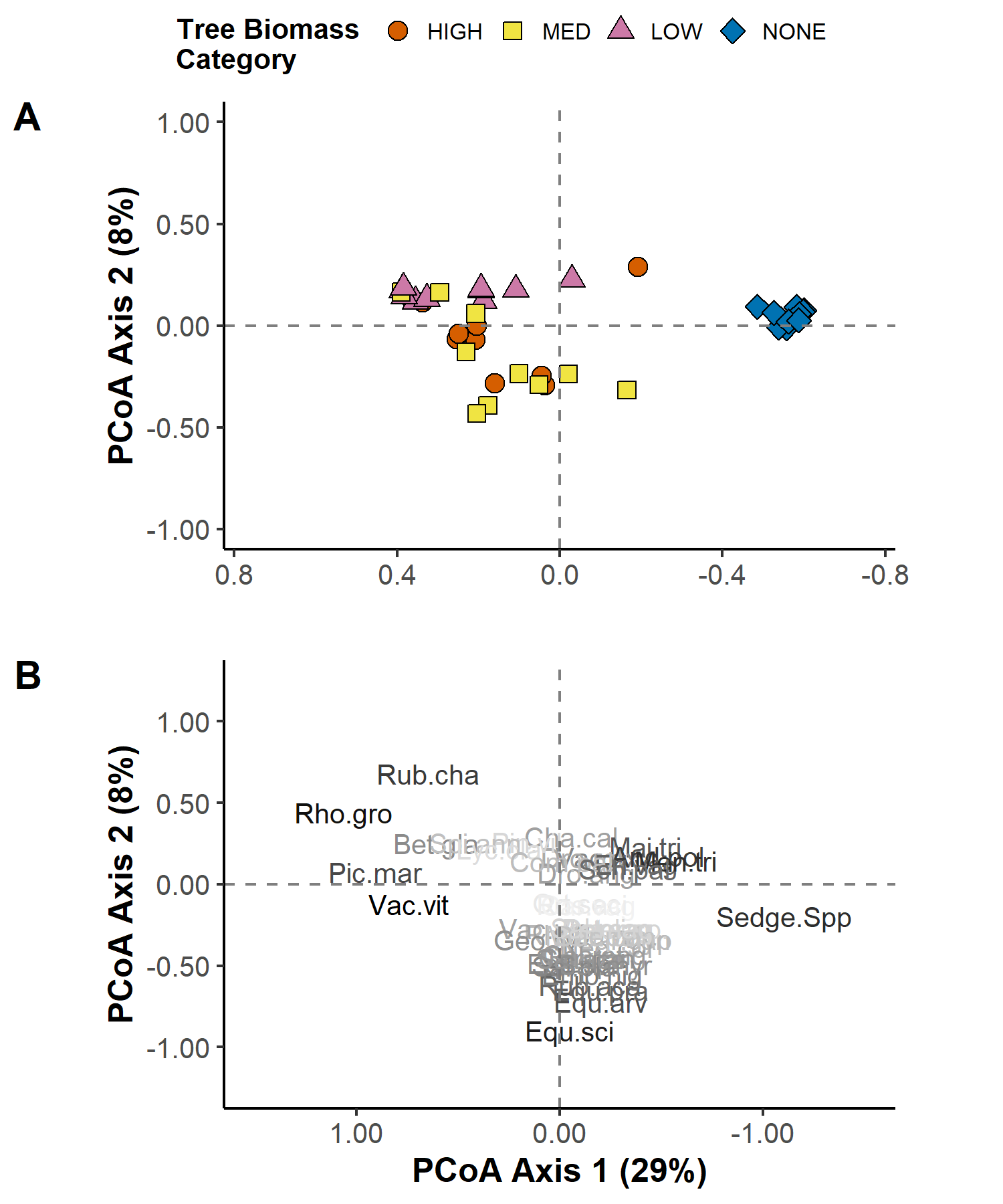

Supplement: Supplementary file 2 — Fig S2 [file ECE3-11-10133-s001.tiff]
