# Supplementary material for: Permafrost condition determines plant community composition and community‐level foliar functional traits in a boreal peatland
Source: Ecol Evol. 2021 Jul 3;11(15):10133–46. doi: 10.1002/ece3.7818 (PMC8328418; doi:10.1002/ece3.7818)
Supplement: Supplementary file 3 — Tab S1‐S6 [file ECE3-11-10133-s003.docx]

## Appendix 1: Supplementary figures and tables


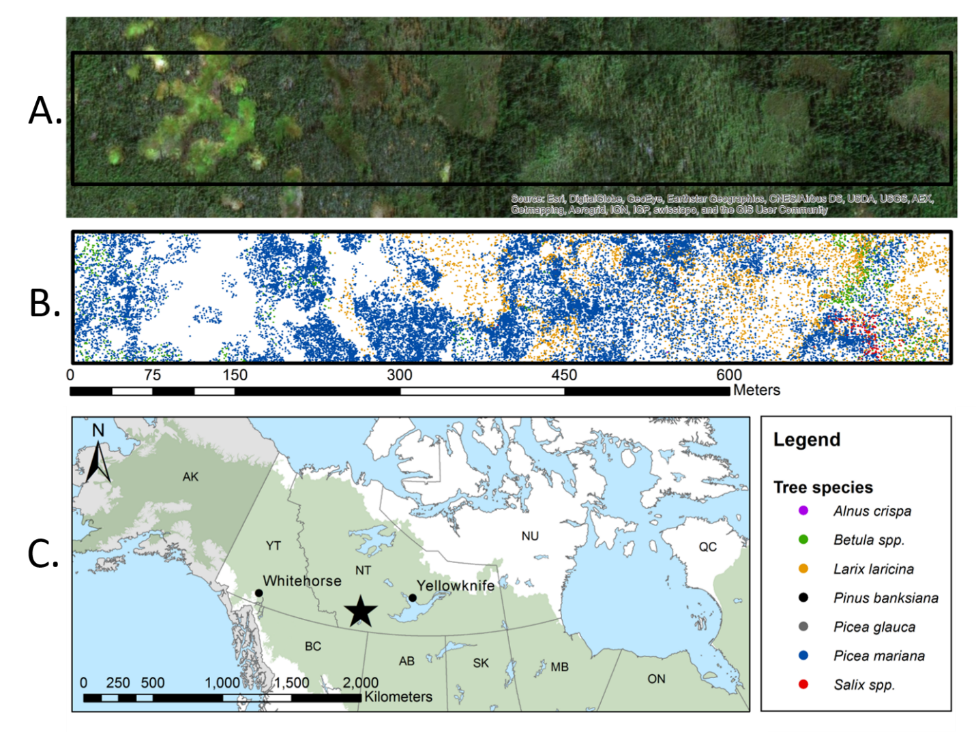


### Figure S1. Images of the aerial view of the Scotty Creek Forest Dynamics plot (FDP) (imagery from: ESRI World Imagery; Esri, DigitalGlobe, GeoEye, i-cubed, USDA FSA, USGS, AEX, Getmapping, Aerogrid, IGN, IGP, swisstopo, and the GIS User Community) (A), stem density of individual species across the FDP (B), and location of the FDP in Canada (C). Map constructed in ArcMap courtesy of K. Dearborn and modified by K. Standen.


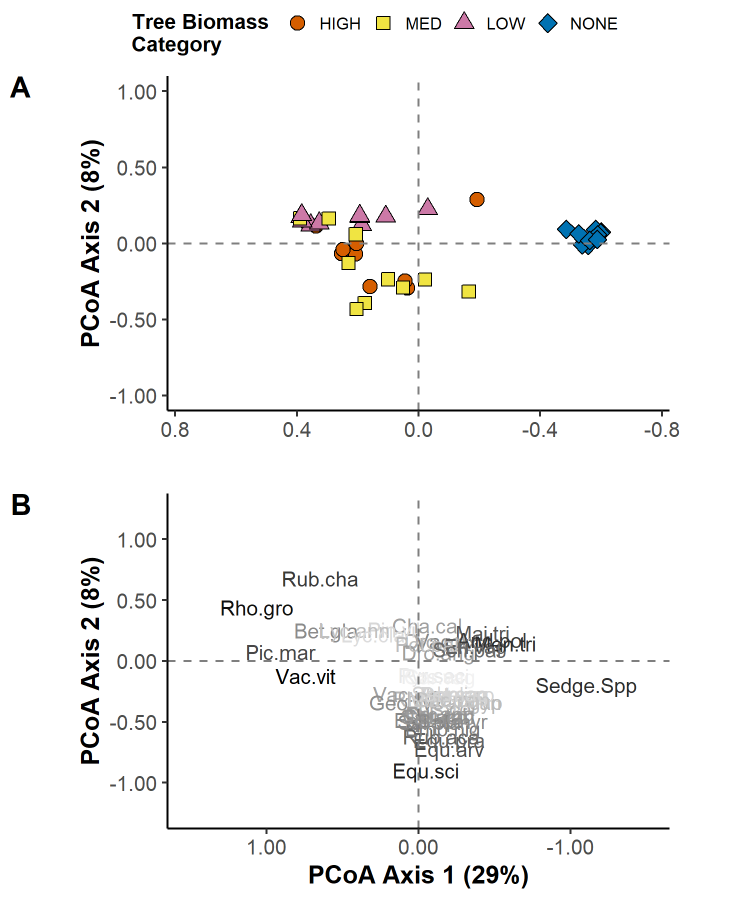


### Figure S2. Principal coordinates analysis of Hellinger transformed stem count data of vascular understory species across Scotty Creek Forest Dynamics Plot showing the site scores (A) and species scores (B).

### Table S1. List of species belonging to several plant function groups sampled for gas exchange across the Scotty Creek Forest Dynamics plot, including their common name, 6 letter acronym and the tree biomass categories in which they were sampled. Note that *Carex* species could not always be identified due to lack of flowers, so we have included all species in the same category.

| **Species** | **Common Name** | **Acronym** | **Tree Biomass**  **Category** |
| --- | --- | --- | --- |
| **Evergreen Shrubs** |  |  |  |
| *Andromeda polifolia* L. | Bog rosemary | And pol | Med, None |
| *Chamaedaphne calyculata* ([L.](https://en.wikipedia.org/wiki/Carl_Linnaeus)) Moench | Leatherleaf | Cha cal | High, Med, Low, None |
| *Rhododendron groenlandicum* ([Oeder](https://en.wikipedia.org/wiki/Georg_Christian_Oeder)) [Kron](https://en.wikipedia.org/wiki/Kathleen_Anne_Kron) & [Judd](https://en.wikipedia.org/wiki/Walter_Stephen_Judd) | Labrador tea | Rho gro | High, Med, Low |
| *Vaccinium vitis-idaea* L. | Lingonberry | Vac vit | High, Med, Low |
| *Vaccinium oxycoccos* L. | Bog cranberry | Vac oxy | High, Med, Low, None |
| **Deciduous Shrubs** |  |  |  |
| *Betula glandulosa* Michx. | Dwarf birch | Bet gla | High, Med, Low |
| *Salix myrtillifolia* Andersson | Blueberry willow | Sal myr | High |
| **Graminoids (sedge)** |  |  |  |
| *Carex* species | Various sedges | Car spp. | High, Med, None |
| **Fern Allies** |  |  |  |
| *Equisetum scirpoides* Michx. | Horsetail | Equ sci | High, Med |
| *Equisetum arvense* L. | Horsetail | Equ arv | High, Med |
| **Forbs** |  |  |  |
| *Geocaulon lividum* (Richardson) Fernald | False toadflax | Geo liv | High |
| *Maianthemum trifolium* ([L.](https://en.wikipedia.org/wiki/Carl_Linnaeus)) [Sloboda](https://en.wikipedia.org/w/index.php?title=Daniel_Sloboda&action=edit&redlink=1" \o "Daniel Sloboda (page does not exist)) | False Solomon’s-seal | Mai tri | None |
| *Menyanthes trifoliata* L. | Buckbean | Men tri | None |
| *Orthilia* secunda ([L.](https://en.wikipedia.org/wiki/Carl_Linnaeus)) House | Wintergreen | Ort sec | High |
| *Rubus chamaemorus* [L.](https://en.wikipedia.org/wiki/Carl_Linnaeus) | Cloudberry | Rub cha | High, Med, Low |
| *Scheuchzeria palustris* L. | Pod grass | Sch pal | None |
| **Coniferous Trees** |  |  |  |
| *Picea mariana* (Mill.) BSP | Black spruce | Pic mar | High, Med, Low |
| *Larix laricina* (Du Roi) K. Koch | Larch | Lar lar | High, Med |

### Table S2. ANOVA table determining the importance of the RDA model (Fig. 2). Significance (p < 0.05) denoted with bold font.

| **Factor** | **df** | **Inertia** | **F** | **p** |
| --- | --- | --- | --- | --- |
| Model | 6 | 0.26 | 4.78 | **<0.01** |
| Residual | 31 | 0.28 |  |  |

### Table S3. ANOVA table for each RDA (Fig. 2) axis demonstrating the importance to interpret each axis. Significant (p < 0.05) axes demonstrated with bold font.

| **RDA Axis** | **df** | **Variance** | **F** | **p** |
| --- | --- | --- | --- | --- |
| 1 | 1 | 0.18 | 20.28 | **<0.01** |
| 2 | 1 | 0.04 | 4.64 | **<0.01** |
| 3 | 1 | 0.02 | 1.76 | 0.52 |
| 4 | 1 | 0.01 | 0.85 | 0.97 |
| 5 | 1 | 0.01 | 0.76 | 0.93 |
| 6 | 1 | <0.01 | 0.37 | 0.97 |
| Residual | 31 | 0.28 |  |  |

### Table S4. ANOVA table for determining significance of each explanatory variable of the RDA (Fig. 2). Significant (p < 0.05) explanatory variables demonstrated with bold font.

| **Explanatory Variable** | **df** | **Variance** | **F** | **p** |
| --- | --- | --- | --- | --- |
| OLT | 2 | 0.19 | 10.33 | **<0.01** |
| Basal Area | 1 | 0.02 | 1.38 | 0.20 |
| FTD | 2 | 0.05 | 2.59 | **0.01** |
| Can. Cover | 1 | 0.01 | 1.42 | 0.20 |
| Residual | 33 | 0.28 |  |  |

### Table S5. ANOVA tables of differences in community-weighted mean functional traits among aboveground tree biomass categories (Fig. 4). Significance denoted by **bold** font.

| **Trait** | **Parameter** | **SS** | **df** | **MS** | **F** | **p** |
| --- | --- | --- | --- | --- | --- | --- |
| **A_mass_** | Tree Biomass | 253.05 | 3 | 84.35 | 5.32 | **0.004** |
|  | Error | 538.80 | 34 | 15.85 |  |  |
| **R_mass_** | Tree Biomass | 13.24 | 3 | 4.42 | 6.03 | **0.002** |
|  | Error | 24.89 | 34 | 0.73 |  |  |
| **N_mass_** | Tree Biomass | 7.66 x 10^6^ | 3 | 2.55 x 10^6^ | 4.50 | **0.009** |
|  | Error | 1.92 x 10^7^ | 34 | 5.67 x 10^5^ |  |  |
| **SLA** | Tree Biomass | 4.56 x 10^6^ | 3 | 1.52 x 10^8^ | 2.85 | *0.052* |
|  | Error | 1.81 x 10^7^ | 34 | 5.33 x 10^7^ |  |  |

### Table S6. ANOVA tables for differences in community-weighted mean functional traits among active layer thickness (ALT) categories (Fig. 6). Significance denoted by **bold** font.

| **Trait** | **Parameter** | **SS** | **df** | **MS** | **F** | **p** |
| --- | --- | --- | --- | --- | --- | --- |
| **A_mass_** | ALT | 299.52 | 2 | 149.76 | 10.05 | **<0.01** |
|  | Error | 491.97 | 33 | 14.91 |  |  |
| **R_mass_** | ALT | 18.46 | 2 | 9.23 | 15.57 | **<0.01** |
|  | Error | 19.56 | 33 | 0.59 |  |  |
| **N_mass_** | ALT | 1.18 x 10^7^ | 2 | 5.89 x 10^6^ | 12.90 | **<0.01** |
|  | Error | 1.51 x 10^7^ | 33 | 4.57 x 10^5^ |  |  |
| **SLA** | ALT | 7.44 x 10^6^ | 2 | 3.72 x 10^6^ | 8.34 | **<0.01** |
|  | Error | 1.47 x 10^7^ | 33 | 4.46 x 10^5^ |  |  |
